# Draft Genome Sequence of *Pseudoalteromonas* sp. Strain A25, a Bacterium with Algicidal Activity against Diatoms

**DOI:** 10.1128/MRA.01254-19

**Published:** 2019-11-07

**Authors:** Hirotaka Kitaguchi, Nanase Masu, Keiko Fujii, Atsushi Mitsutani

**Affiliations:** a Department of Marine Bio-Science, Fukuyama University, Fukuyama, Hiroshima, Japan; b Graduate School of Technology, Fukuyama University, Fukuyama, Hiroshima, Japan; Georgia Institute of Technology

## Abstract

Pseudoalteromonas sp. strain A25 is a marine bacterium that reveals strong algicidal activity against diatoms. We report the draft genome sequence for this strain, which consists of two contigs (3,887,303 bp and 850,536 bp). Further genomic analysis might facilitate an understanding of the algicidal mechanisms of this strain.

## ANNOUNCEMENT

Harmful algal blooms (HABs) can cause serious economic loss in fisheries and aquaculture (1), so applicable methods for controlling HABs using algicidal bacteria, fungi, and viruses have attracted much attention (2). Pseudoalteromonas sp. strain A25 is a naturally occurring, Gram-negative, rod-shaped, and yellow-pigmented-colony-forming marine bacterium that was isolated from seawater of the Ariake Sea, Japan, in 1994 (3). This strain has a strong algicidal activity against diatoms (3). In a previous study, some of the proteins induced in the stationary phase of this bacterium might be involved in the algicidal process (4), but the details of the algicidal process and the algicidal compounds produced are still unknown. Genomic analysis of this strain is expected to contribute to the understanding of the algicidal mechanism.

*Pseudoalteromonas* sp. strain A25 from our private collection was cultured at 15°C with rotary shaking (200 rpm) in a modified SWM-III medium (5) with 0.1% Casitone (Difco) and 0.05% yeast extract (Difco). We prepared genomic DNA from this strain using a DNeasy blood and tissue kit (Qiagen GmbH). The genome of *Pseudoalteromonas* sp. strain A25 was sequenced using the NextSeq 500 system (Illumina, Inc.) and GridION X5 (Oxford Nanopore Technologies Ltd.). For NextSeq sequencing, genomic DNA was sheared with a Covaris S2 sonicator to obtain ∼500-bp DNA fragments. A library was prepared from 500 ng of fragmented DNA using a preparation kit (HyperPrep kit; Kapa Biosystems) and then sequenced with NextSeq technology to produce 2 × 151-bp paired-end reads. A total of 1,107,371 reads with a Q30 of 81.8% were obtained by NextSeq sequencing. For GridION analysis, 1,000 ng of genomic DNA was barcoded using the native barcoding expansion kit (Oxford Nanopore Technologies Ltd.). A library was prepared using a ligation sequence kit (SQK-LSK109). By GridION analysis, 1,551,268 reads (average length, 3149.6 bp) were obtained. NextSeq reads were trimmed using the parameters -q 20 -l 127 with Sickle v1.33 (6). A total of 984,910 filtered reads were used for subsequent assembly. Adaptor sequences in the reads from GridION were trimmed with Porechop v0.2.3, and the trimmed reads were quality filtered using the parameters –min_mean_q 8 –min_length 1,000 with Filtlong v0.2.0. Error-prone read data from GridION were processed using Canu v1.8 (7). A total of 1,216,742 reads were used for the subsequent assembly. Finally, hybrid assembly of NextSeq and GridION reads was done using Unicycler v0.4.7 (8). Default parameters were used for all software, unless otherwise noted.

The draft genome includes two contigs with a total size of 4,737,841 bp. Unicycler specified that these two contigs were circular. The GC content is 42.1%. The *N*_50_ contig and longest contig sizes are the same (3,887,305 bp). The assembled genome was annotated using the DDBJ Fast Annotation and Submission Tool (DFAST) pipeline with standard settings (9). Annotation detected 3,947 coding sequences and 89 tRNA genes.

### Data availability.

The draft genome sequence of *Pseudoalteromonas* sp. A25 was deposited in DDBJ/ENA/GenBank under accession no. AP021846 and AP021847. The raw sequencing reads were submitted to the DRA under accession no. DRA008772.
